# Visual Object Tracking Using Structured Sparse PCA-Based Appearance Representation and Online Learning

**DOI:** 10.3390/s18103513

**Published:** 2018-10-18

**Authors:** Gang-Joon Yoon, Hyeong Jae Hwang, Sang Min Yoon

**Affiliations:** 1 National Institute for Mathematical Science, 70 Yuseong-daero 1689 beon-gil, Yuseong-gu, Daejeon 34047, Korea; gangjoon@gmail.com; 2 Artificial Intelligence Research Institute, 22, Daewangpangyo-ro 712beon-gil, Bundang-gu, Seongnam-si 463400, Gyeonggi-do, Korea; ghkdgudwo@kookmin.ac.kr; 3 College of Computer Science, Kookmin University, 77 Jeongneung-ro, Seongbuk-gu, Seoul 02707, Korea

**Keywords:** visual object tracking structured sparse PCA, appearance model, online learning, structured visual dictionary

## Abstract

Visual object tracking is a fundamental research area in the field of computer vision and pattern recognition because it can be utilized by various intelligent systems. However, visual object tracking faces various challenging issues because tracking is influenced by illumination change, pose change, partial occlusion and background clutter. Sparse representation-based appearance modeling and dictionary learning that optimize tracking history have been proposed as one possible solution to overcome the problems of visual object tracking. However, there are limitations in representing high dimensional descriptors using the standard sparse representation approach. Therefore, this study proposes a structured sparse principal component analysis to represent the complex appearance descriptors of the target object effectively with a linear combination of a small number of elementary atoms chosen from an over-complete dictionary. Using an online dictionary for learning and updating by selecting similar dictionaries that have high probability makes it possible to track the target object in a variety of environments. Qualitative and quantitative experimental results, including comparison to the current state of the art visual object tracking algorithms, validate that the proposed tracking algorithm performs favorably with changes in the target object and environment for benchmark video sequences.

## 1. Introduction

Visual object tracking systems have gained continuous attention and focus in the area of computer vision and pattern recognition because they can be applied to various fields, such as robotics, video surveillance, user-centered interaction systems, video communication and compression and augmented reality [1,2,3,4]. A large number of tracking algorithms has been proposed to follow the moving object in a given image sequence, while simultaneously keeping track of target identities through the significant pose changes, illumination variations and occlusions by focusing on finding appearance and motion models. To evaluate the performance of the state of the art visual object tracking methodologies quantitatively and qualitatively, benchmark tests [5,6] were conducted using a large database including ground-truth object positions to understand how these algorithms perform and effectively analyze algorithm advances.

Most state of the art visual object tracking algorithms with reported benchmark tests were formulated using the Bayesian framework [7] where the maximum a posteriori (MAP) state of the given observation was estimated by decomposing the visual object tracking system into three components.

1. An appearance model that captures the visual characteristics of the target object and evaluates the similarity between observed samples and the model.
2. A motion model that locates the target between successive frames utilizing certain motion hypotheses.
3. An optimization strategy that associates the appearance model with the motion model and finds the most likely location in the current frame.

In the Bayesian visual object tracking framework, the main issue of robust target object tracking is to find models for status and observation, such as target representation and localization, as well as filtering and data association. Target object representation and localization methodologies follow a bottom-up process that provides a variety of tools for identifying the moving object. The specific strategy for successfully locating and tracking the target object depends on features in the color, appearance and time spaces. Filtering and data association are mostly top-down processes, incorporating prior information about the scene or object, dealing with object dynamics and evaluating different hypotheses.

The core technique of visual object tracking in the Bayesian framework aims to robustly estimate the motion state of a target object with a defined appearance model in each frame from given image sequences. To achieve visual object tracking, it is necessary to categorize the appearance model into several task-specific categories. Popular appearance models used in object tracking can be separated into global and local visual appearance models [8]. Global visual representation of the target object is simple and computationally efficient for fast object tracking, but is very sensitive to target deformation and environmental changes, including illumination. A multi-cue strategy is adopted in relation to the global features, incorporating multiple visual information types, to deal with complicated appearance changes. In contrast, local visual appearance representation is robust to global appearance change by capturing the local structural object appearance. However, the representation often suffers from noise distribution and background distraction.

Sparse representation and dictionary learning for online appearance modeling have been recently proposed as an alternative solution, formulating the over-complete dictionary as a linear combination of basis functions. However, global linear sparse representation has problems with partial occlusion and local deformation. Since the dictionary uniformly emphasizes the object, occlusion and local deformation can be seen as noise when estimating similarity [9,10,11]. Another characteristic inherent in natural images is their high dimensionality, which causes complex and expensive computation. Exploration of the specific structure of sparsity as a prior enables dictionary learning to reduce computational costs effectively [12,13,14]. Therefore, we propose a structured sparse principal component analysis (PCA)-based subspace representation to represent the appearance model of the target object effectively and online learning techniques for robust visual object tracking. We use the structured sparse PCA to find a sparse linear combination over a basis library containing target and trivial templates by reducing the data dimension. The proposed structured sparse PCA-based visual object tracking within the Bayesian framework is decomposed into initialization, observation model, motion tracking model and update. The structured spare PCA-based appearance model representation and learning of domain-specific over-complete dictionaries are used to obtain MAP dictionary estimates within an appropriately chosen dictionary. The main contributions of our proposed robust visual object tracking system are as follows.

- Structured sparse PCA-based appearance representation and learning for efficient description of the target object with few dictionary entries, to reduce the high-dimensional descriptor and to retain the structure.
- Local structure enforced similarity measures to avoid problems from partial occlusion, illumination and background clutter.
- Training image selection for robust online dictionary learning and updating by considering the probability that the training image contains the target, as opposed to the existing methods that choose the most recent training images.

Section 2 reviews relevant previous visual object tracking approaches, and Section 3 details tracking target objects from a given image by modeling the observation and motion using the proposed structured sparse PCA-based representation within the Bayesian framework. Section 4 quantitatively and qualitatively compares the proposed and current state of the art approaches experimentally. Section 5 summarizes the outcomes, concludes the paper and discusses future work.

## 2. Review of Previous Related Work

There is a rich literature in visual object tracking methodologies dealing with target object representations, search mechanisms and model updating. Sparse representation and modeling also have a fruitful literature exploiting prior information within the predefined structure of the basis library and contiguous spatial distribution of deformable target objects. We review some of the important milestones in terms of visual object tracking and sparse representation-based modeling.

### 2.1. Visual Object Tracking System

Many tracking methods have been proposed, largely separated into generative and deterministic methods. Generative visual object tracking methods search for the most similar region to the target object within a neighborhood, whereas discriminative methods treat tracking as a binary classification problem and aim to design a classifier to distinguish the target object from the background [15].

Early visual object tracking systems focused on generative methods, such as the Lucas–Kanade tracker [16], Kalman filter [17,18] and mean-shift (MS) tracker [19,20]. The Kalman filter [17] used for visual object tracking commonly uses the state and observation model uncertainties to calculate actual Gaussian noise, which causes certain parameter estimations to produce errors in the model, with consequent decreased estimation precision. The particle filter (PF) is efficient for conventional tracking problems with non-Gaussian distributions and multi-modality [21]. MS-based approaches are efficient for tracking non-rigid objects whose appearances are defined by histograms, but this makes them poor at dealing with illumination and/or pose variations [19,20].

Multiple instance learning (MIL)-based tracking [22] implements discriminative tracking by building a boosting classifier that tracks bags of image patches by incrementally updating the training patches over time. Online appearance learning (OAL)-based visual object tracking uses different target object appearances as a set of probability mass functions to adaptively deal with pose variations [23]. Many approaches attempted to efficiently represent the variation of rigid or limited deformation motion using an adaptive appearance model, such as incremental visual [24] and fragment-based (Frag) [25] trackers. Kelal et al. [26] proposed a paradigm for training a binary classifier from labeled and unlabeled examples called P-N learning for visual object tracking. Tracking-learning-detection (TLD) is an award-winning, real-time algorithm for tracking unknown objects in video streams that simultaneously tracks the object, learns its appearance and detects it whenever it appears in the video [27]. Struct [28] is an extended version of TLD using kernels. On the other hand, sparse representation-based visual object tracking systems like sparse collaborative appearance (SCM) [29], visual tracking decomposition (VTD) [30], the sparse representation-based $l_{1}$ tracker [31], the structured sparse tracking (SST) [32] model and sparse mask models [33,34] use an appearance model to find the sparsest linear combination of basis functions from an over-complete dictionary. However, most dictionary learning-based systems still have problems in high-dimensional reduction. Deep learning-based machine learning techniques have been recently applied to separate target objects from target candidate image templates [35,36,37,38,39] and showed a good performance to track the target object, but this requires numerous training templates.

In contrast to visual tracking approaches based on pixel-based observation models, superpixel tracking (SPT) [40] uses middle level features to both remove noise and enforce the target object color of the candidate template.

### 2.2. Sparse Representation-Based Learning

Sparse signal representation is an extremely powerful tool for acquiring, representing and compressing high dimensional signals. Mathematically, solving a sparse representation and learning involves seeking the sparsest linear combination of basis functions from an over-complete dictionary. The basic concept of how to represent or reconstruct signals with sparse samples is an extremely important problem in many practical fields, such as signal processing, machine learning, computer vision and robotics. Compressive sensing (CS) is based on the principle that signal sparsity can be exploited to recover the original signal from significantly less samples than required by the Shannon–Nyquist theorem [41,42]. Generally, CS algorithms include three basic components: sparse representation, encoding measuring and a reconstruction [12]. In particular, sparse representation that approximately solves a system of equations with sparse vectors is popularly applied for pattern recognition because it exploits a linear combination of training samples to represent the test sample and computes sparse representation coefficients of the linear representation system [43,44,45].

Structured sparse representation is an extension of standard sparse representation in statistical signal processing and learning [46,47]. Motivated by potential group structures on feature sets, group sparse representation has become popular in recent years. Group sparsity is used not only for estimating hyper-parameters in the sparse prior model, but also for group least absolute shrinkage and selection operator (LASSO). Techniques using strong group for group LASSO have been developed and show superior performance for strongly group-sparse feature sets [48]. However, group LASSO works well only under the strong group sparsity assumption and does not apply for more general structures, such as overlapping groups, and tonal or transient structures. Therefore, Huang et al. [14] proposed that sparse representation can be solved by a structured greedy algorithm when a coding scheme can be approximated by block coding with base blocks.

## 3. Structured Sparse PCA-Based Tracking and Online Dictionary Learning

**Figure 1:**
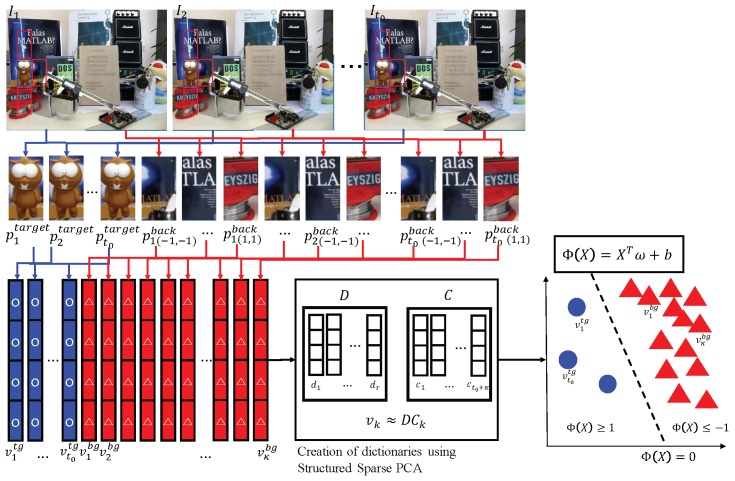
Representation of the target object using structured sparse PCA and deterministic classification between the target object and background image patches.

For visual object tracking, it is reasonable to assume that the object trajectory is continuous and object features are consistent or change insignificantly over a short time interval. Thus, once a representation of the feature vector is found in terms of fix-ahead dictionaries, consecutive representations of the feature vectors are almost constant. Therefore, we propose an object tracking method by classifying the target appearance model’s coefficients. The dictionaries are generated from appearance features by applying structured sparse PCA and updated using the last data. The object tracking comprises three modes: observation, tracking and update within the Bayesian framework, as shown in Figure 1.

### 3.1. Notations and Symbols

Before proceeding to the technical details, we introduce the notations and symbols used throughout this paper, as shown in Table 1. Lower case letters denote real variables, and upper case (capital) letters denote multi-dimensional variables, such as images and matrices, except for the case $Y_{t}$, which denotes an observation random variable taking real numbers. Column vectors given are shown as boldface, and mappings are denoted by letters of the Greek alphabet.

### 3.2. Bayesian Framework-Based Visual Object Tracking

The traditional visual object tracking algorithm can be formulated with the Bayesian framework where the maximum a posteriori (MAP) estimation of the state given the observations up to time *t* is expressed as:

(1) $$\begin{array}{cc} p(X_{t}|Y_{1:t}) & =\frac{p\left(Y_{t}|Y_{1:t-1},X_{t}\right)p\left(X_{t}|Y_{1:t-1}\right)}{p(Y_{t}|Y_{1:t-1})}\\ & =\frac{p(Y_{t}|X_{t})}{n_{t}}\int p\left(X_{t}|X_{t-1}\right)p\left(X_{t-1}|Y_{1:t-1}\right)dX_{t-1}, \end{array}$$

where $X_{t}$ is the state at *t*; $Y_{1:t}$ denotes all the observations up to *t*; and $n_{t}$ is a normalization term,

(2) $$\begin{array}{c} n_{t}=p\left(Y_{t}|Y_{1:t-1}\right)=\int p\left(X_{t}|Y_{1:t-1}\right)p\left(Y_{t}|Y_{1:t-1},X_{t}\right)dX_{t}. \end{array}$$

We use the following assumptions.

(i) State $X_{t}$ is independent of the past given the present $X_{t-1}$,
  (3) $$\begin{array}{c} p\left(X_{t}|X_{1:t-1},Y_{1:t-1}\right)=p\left(X_{t}|X_{t-1}\right). \end{array}$$
(ii) Observations $Y_{1:t}$ are conditionally independent given $X_{t}$,
  (4) $$\begin{array}{c} p\left(Y_{t}|Y_{1:t-1},X_{t}\right)=p\left(Y_{t}|X_{t}\right). \end{array}$$

We also employed the Chapman–Kolmogorov equation for Equation (1),

$$p\left(X_{t}|Y_{1:t-1}\right)=\int p\left(X_{t}|X_{t-1}\right)p\left(X_{t-1}|Y_{1:t-1}\right)dX_{t-1}.$$

In the visual object tracking scheme, the target state is defined as $X_{t}=\left({\vec{x}}_{t}^{c},w_{t}^{sx},h_{t}^{sy}\right)$, where ${\vec{x}}_{t}^{c}$ represents the center location of the target and $w_{t}^{sx}$ and $h_{t}^{sy}$ denote its scale in the *x* and *y* directions, respectively. In terms of observation, we need to construct an effective observation model $p(Y_{t}|X_{t})$ and an efficient motion model $p(X_{t}|X_{t-1})$. The state estimate of the target $X_{t}$ at time *t* can be obtained by the MAP estimate over the *M* samples $X_{t}^{j}$ and its measurements $Y_{t}^{j}$ for j=1,…,M, given $X_{t-1}$,

(5) $$X_{t}=\underset{X_{t}^{j}}{\mathrm{argmax}}p\left(X_{t}^{j}|Y_{t}^{j},X_{t-1}\right).$$

It is worth noting that even though we need the measurement quantities $p(Y_{t}^{j}|X_{t-1})$ in solving the optimization (5) from Bayes’ rule p(x|y,z)=p(y|x,z)p(x|z)/p(y|z), we may regard the denominator as a constant for all j=1,…,M and solve the maximization by finding the maximum of likelihood times prior as given in (13). This is because given $X_{t}$, the measurements (evidence) $Y_{t+1}$ and $Y_{t}$ for the two consecutive targets $X_{t+1}$ and $X_{t}$ remain the same. We shall see this precisely in Section 3.4.

Based on the MAP estimation, we decompose the visual object tracking procedure into:

1. structured sparse PCA-based observation and appearance representation using deterministic target object separation from background patch images,
2. motion tracking and
3. online update.

### 3.3. Deterministic Modeling Using Structured Sparse PCA-Based Appearance Representation

To construct the dictionary from the $t_{0}$ initial image sequences, we extract image patches using windows surrounding the target object for each $t=1,\ldots ,t_{0}$. Figure 1 shows the proposed procedure to separate the target object and background image patches around the target object, representing appearances using structured sparse representation. Let us explain the learning mode of the target object tracking in more detail. We create tracking dictionary vectors ${\left\{{\vec{d}}_{i}\right\}}_{i=1}^{r}$ by applying feature descriptors extracted from observation frames $I_{1:t_{0}}$ to the structured sparse PCA algorithm as follows.

1. We take the same sized image patches ${\left\{{\vec{p}}_{t}^{target}\right\}}_{t=1}^{t_{0}}$ centered at $\left(x_{t}^{c},y_{t}^{c}\right)$ from frames $I_{1:t_{0}},$ respectively.
  Recall that states $X_{t}=\left({\vec{x}}_{t}^{c},w_{t}^{sx},h_{t}^{sy}\right)$ consist of the center location ${\vec{x}}_{t}^{c}=\left(x_{t}^{x},y_{t}^{c}\right)$ of the target and its window size $\left(w_{t}^{sx},h_{t}^{sy}\right)$ in the *x* and *y* directions, respectively. From each patch ${\vec{p}}_{t}^{target},$ $t=1,\ldots ,t_{0},$ we construct the descriptor ${\vec{v}}_{t}^{tg}\in R^{s}$ of the target object by sequentially accumulating gradient histograms from equally-divided subregions of ${\vec{p}}_{t}^{target}$.
  To enhance tracking performance, we also create background feature descriptors ${\vec{v}}_{j}^{bg}\in R^{s}$ from the four background patch images $\left\{{\vec{p}}_{t,(a_{x},b_{y})}^{back}\in I_{t}|a_{x},b_{y}=1,-1\;\;\mathrm{and}\;\;a_{x}^{2}+b_{y}^{2}=1,\;t=1,\ldots ,t_{0}\right\}$ around the target patch ${\vec{p}}_{t}^{target}$ as follows.
  - For each $t=1,\ldots ,t_{0}$, patches ${\vec{p}}_{t,(a_{x},b_{y})}^{back}$ are subimages of $I_{t}$ centered at $\left(x_{t}^{c}+a_{x}w_{t}^{sx},y_{t}^{c}+b_{y}h_{t}^{sy}\right)$ with the same size as ${\vec{p}}_{t}^{target}$.
  - When the domain of ${\vec{p}}_{t,(a_{x},b_{y})}^{back}$ does not entirely belong to that of $I_{t}$, we regard it as an empty set.
  - Let ${\left\{{\vec{v}}_{j}^{bg}\right\}}_{j=1}^{\kappa }\in R^{s}$ with $\kappa \leq 4t_{0}$ be background appearance descriptors obtained from background patches ${\vec{p}}_{t,(a_{x},b_{y})}^{back}$ in the same manner used to create the target descriptors.
2. After creating the appearance feature descriptors ${\vec{v}}_{t}^{tg}$ and ${\vec{v}}_{j}^{bg}$, we apply the constrained structured sparse PCA dictionary learning algorithm to the target and background descriptors to find dictionaries ${\left\{{\vec{d}}_{i}\right\}}_{i=1}^{r}\in R^{s}$,
  (6) $$\begin{array}{cc} \left(D,C\right) & =\underset{\begin{array}{c} D\in R^{s\times r}\\ C\in R^{r\times (t_{0}+\kappa )} \end{array}}{\mathrm{argmin}}H\left(D,C\right)\\ & \mathrm{subject}\;\mathrm{to}\;\;\;{∥{\vec{c}}_{j}∥}_{2}\leq 1,\;j=1\ldots ,t_{0}+\kappa , \end{array}$$
  where the objective function H(D,C) is given by:
  $$H\left(D,C\right)=\frac{1}{2s(t_{0}+\kappa )}{∥V-DC∥}_{F}^{2}+\lambda \sum_{i=1}^{r}\Omega_{\nu }\left({\vec{d}}_{i}\right)$$
  and $V={\left({\vec{v}}_{i}\right)}_{i=1}^{t_{0}+\kappa }$ is the $s\times (t_{0}+\kappa )$ matrix with ${\vec{v}}_{1:t_{0}}^{tg}$ and ${\vec{v}}_{1:\kappa }^{bg}$ column vectors; $D={\left({\vec{d}}_{i}\right)}_{i=1}^{r}\in R^{s\times r}$ is the dictionary matrix; and $C={\left({\vec{c}}_{i}\right)}_{i=1}^{t_{0}+\kappa }\in R^{r\times (t_{0}+\kappa )}$ is the coefficient matrix, such that for $i=1,\ldots ,t_{0}+\kappa$, ${\vec{v}}_{i}$ is (approximately or exactly) expressed by a linear combination of ${\vec{d}}_{j}$ with coefficients ${\vec{c}}_{i}={\left(c_{ji}\right)}_{j=1}^{r}$,
  $${\vec{v}}_{i}\approx \sum_{j=1}^{r}c_{ji}{\vec{d}}_{j}=D{\vec{c}}_{i},\;{\vec{c}}_{i}={\left(c_{1i},c_{2i},\ldots ,c_{ri}\right)}^{T}$$
  for $i=1,\ldots ,t_{0}+\kappa .$
3. Let ${∥\cdot ∥}_{F}$ be the Frobenius matrix norm, ${∥A∥}_{F}^{2}=trace\left(AA^{T}\right)=\sum_{i=1}^{n}\sum_{j=1}^{m}a_{ij}^{2}$, for $A=\left(a_{ij}\right)\in R^{n\times m}$; ${∥\cdot ∥}_{2}$ the Euclidean norm; and $\Omega_{\nu }$ a quasi-norm that controls the sparsity and structure of the support of ${\vec{d}}_{j}.$ In this work, the quasi-norm $\Omega_{\nu }$ is defined as follows. Let $G_{1},G_{2},G_{3},G_{4}$ be four mutually disjoint subsets of {1,2,…,s}. Then, every vector $\vec{d}=\left(d_{1},\ldots ,d_{s}\right)\in R^{s}$ is decomposed into four subvectors ${\vec{d}}_{k}=\left(d_{1}^{k},\ldots ,d_{s}^{k}\right),k=1,2,3,4$ such that for 1≤k≤4 and 1≤j≤s,
  $$d_{j}^{k}=\left\{\begin{array}{cc} d_{j}, & \mathrm{if}\;j\in G_{k}\\ 0, & \mathrm{otherwise} \end{array}\right.$$
  Then, $\Omega_{\nu }\left(\vec{d}\right)$ is defined as:
  $$\Omega_{\nu }\left(\vec{d}\right)={\left({∥{\vec{d}}_{1}∥}_{2}^{\frac{1}{2}}+{∥{\vec{d}}_{2}∥}_{2}^{\frac{1}{2}}+{∥{\vec{d}}_{3}∥}_{2}^{\frac{1}{2}}+{∥{\vec{d}}_{4}∥}_{2}^{\frac{1}{2}}\right)}^{2}.$$
  We refer to [49] and the references therein for details on the quasi-norm. The decomposition of *V* into DC enables us to reduce the dimensionality of the descriptors using Equation (6).
  Although there is clearly a limitation in representing high dimensional descriptors using a smaller number of vectors than the dimension, the proposed structured sparse PCA is more effective to represent nonlinear and high dimensional descriptors by reducing the dimension while retaining the target object structure. For more details of structured sparse PCA algorithms, refer to the original paper [49].
4. Finally, we find a linear support vector machine (SVM) $\Phi :R^{s}\to R$, such that $\Phi \left({\left(DC\right)}_{i}\right)\geq 1\;\;\left(i=1,\ldots ,t_{0}\right)$ for the target feature-related column vectors of DC and $\Phi \left({\left(DC\right)}_{i}\right)\leq -1\;\;\left(i=t_{0},\ldots ,t_{0}+\kappa \right)$ for the background appearance feature related column vectors of DC, where ${\left(DC\right)}_{i}$ denotes the *i*-th column vector of DC, i.e., ${\left(DC\right)}_{i}=D{\vec{c}}_{i}.$ Using the classifier Φ, we estimate observation $Y_{t}\in \left\{1,-1\right\}$ as:
  (7) $$Y_{t}=\left\{\begin{array}{ccc} 1 & (\mathrm{target}), & \mathrm{if}\;\Phi ({\vec{v}}_{t}^{tg})\geq 0\\ -1 & (\mathrm{background}), & \mathrm{otherwise} \end{array}\right.,$$
  where we recall that ${\vec{v}}_{t}^{tg}$ is the target feature descriptor obtained from state $X_{t}$. Note that when the target object is occluded or not observed, the value of the observation becomes negative.

The procedure of deterministic separation using the structured sparse PCA-based representation of the target and the background is shown in Algorithm 1.

**Algorithm 1:** Discriminative classification of target objects. **Input**: frame images $I_{1:t_{0}}$, states $X_{1:t_{0}}$, integers $r_{tg},r_{bg}>0$
 1.take target patches ${\vec{p}}_{1:t_{0}}^{target}$ 2.take background patches ${\vec{p}}_{t,(a_{x},b_{y})}^{back},\;t=1,\ldots ,t_{0}$ 3.create target appearance descriptors ${\vec{v}}_{1:t_{0}}^{tg}\in R^{s}$ 4.create background appearance descriptors ${\vec{v}}_{1:\kappa }^{bg}\in R^{s}$ 5.find (D,C) by applying structured sparse PCA (6) 6.find optimized classifier Φ such that $\Phi ({\left(DC\right)}_{i})\geq 1\;$for $i=1,\ldots ,t_{0}$ and $\Phi ({\left(DC\right)}_{i})\leq -1$ for $i=t_{0},\ldots ,t_{0}+\kappa$ **Output**: target appearance descriptors ${\vec{v}}_{1:t_{0}}^{tg}\in R^{s}$ and classifier Φ

### 3.4. Motion Tracking Model and Online Update

Using the learned dictionary of the target object and classifier, we track the target object for frames ${\left\{I_{t+1}\right\}}_{t+1>t_{0}}$ from the previous states $X_{t}$. The motion model $p(X_{t+1}|X_{t})$ starts from the Gaussian assumption:

(8) $$\begin{array}{c} p\left(X_{t+1}|X_{t}\right)=N\left(X_{t+1};X_{t},\vec{\sigma }\right)\\ =\frac{1}{{\left(2\pi \right)}^{2}{\left|\vec{\sigma }\right|}^{1/2}}\exp\left(-\frac{1}{2}{\left(X_{t+1}-X_{t}\right)}^{T}{\vec{\sigma }}^{-1}\left(X_{t+1}-X_{t}\right)\right), \end{array}$$

where $\vec{\sigma }$ is a diagonal covariance matrix whose elements are the standard deviations for location and size and $|\vec{\sigma }|$ is the determinant of $\vec{\sigma }.$

Let $I_{t+1}$ be the frame at $t+1>t_{0}$, and assume we already have states $X_{1:t}.$ We randomly take *M* candidate states ${\left\{{\hat{X}}_{t+1}^{j}\right\}}_{j=1}^{M}$ around $\left(x_{t}^{c},y_{t}^{c}\right)$ in $I_{t+1}$ with ${\hat{X}}_{t+1}^{j}∼N\left(X_{t},\vec{\sigma }\right)$. Similar to the observation mode, we build the *M* descriptors ${\left\{{\vec{v}}_{t+1}^{j}\right\}}_{j=1}^{M}$ from sample states ${\left\{{\hat{X}}_{t+1}^{j}\right\}}_{j=1}^{M}$.

Since the observation model $p(Y_{t}|X_{t})$ with given state $X_{t-1}$ implies the confidence of an observation $Y_{t}$ at state $X_{t}$ being the target, the likelihood $p(Y_{t+1}|{\hat{X}}_{t+1}^{j},X_{t})$ is proportional to its confidence:

$$p\left(Y_{t+1}|{\hat{X}}_{t+1}^{j},X_{t}\right)\propto \omega \left(Y_{t+1}|{\hat{X}}_{t+1}^{j},X_{t}\right).$$

Given the target state $X_{t}$ at time t, the confidence $\omega (y|X_{t+1},X_{t})$ for the target candidates $X_{t+1}$ with positive confidence value increases as we observe the targets in a larger area, whereas confidence for target candidates with negative confidence decreases. Therefore, we evaluate confidence $\omega (y|X_{t+1},X_{t})$ comparing with state $X_{t}$ as:

(9) $$\omega \left(y|X_{t+1},X_{t}\right)=\frac{1}{1+e^{-y\Phi ({\vec{v}}_{t+1})}}\cdot {\left(\frac{w_{t+1}^{sx}+h_{t+1}^{sy}}{w_{t}^{sx}+h_{t}^{sy}}\right)}^{y},$$

where y=1,−1 and ${\vec{v}}_{t+1}$ is the feature descriptor extracted from the target state $X_{t+1}$ and $w_{t+1}^{sx}\cdot h_{t+1}^{sy}$ denotes the window size of $X_{t+1}$. We note that in the tracking mode, we estimate the observation in (7) and the confidence in (9) by applying the descriptor $\vec{v}$ directly to the SVM, $\Phi (\vec{v}),$ instead of using the dictionary representation ${\left(D^{T}D\right)}^{-1}D^{T}\vec{v}$ as we construct the SVM Φ in the initialization mode. This is because the descriptor $\vec{v}$ and its dictionary representation ${\left(D^{T}D\right)}^{-1}D^{T}\vec{v}$ are much similar for ${\left(D^{T}D\right)}^{-1}D^{T}\vec{v}$, which minimizes $∥\vec{v}-D\vec{w}{∥}_{2}$, so that it is cheaper to apply the descriptor to SVM rather than to utilize the representation, which requires the computation of the inverse matrix ${\left(D^{T}D\right)}^{-1}.$

Now, the likelihood $p(Y_{t+1}|{\hat{X}}_{t+1}^{j},X_{t})$ of $Y_{t+1}$ given states ${\hat{X}}_{t+1}^{j}$ and $X_{t}$ is defined as:

(10) $$p\left(Y_{t+1}|{\hat{X}}_{t+1}^{j},X_{t}\right)=\frac{1}{n_{\omega }}\omega \left(Y_{t+1}|{\hat{X}}_{t+1}^{j},X_{t}\right),$$

for j=1,2,…,M with the normalizing factor $n_{\omega }=\omega \left(-1|{\hat{X}}_{t+1}^{j},X_{t}\right)+\omega \left(1|{\hat{X}}_{t+1}^{j},X_{t}\right).$ Applying the motion model $p({\hat{X}}_{t+1}^{j}|X_{t})$ obtained from Equation (8) and the observation model $p(Y_{t+1}|{\hat{X}}_{t+1}^{j},X_{t})$ obtained from Equation (10) to the Bayesian formulation in Equation (1), we estimate the a posteriori probability $p({\hat{X}}_{t+1}^{j}|Y_{t+1},X_{t})$ as:

(11) $$\begin{array}{cc} p({\hat{X}}_{t+1}^{j}|Y_{t+1},X_{t}) & =\frac{p\left(Y_{t+1}|{\hat{X}}_{t+1}^{j},X_{t}\right)p\left({\hat{X}}_{t+1}^{j}|X_{t}\right)}{p(Y_{t+1}|X_{t})}\\ & =\frac{p\left(Y_{t+1}|{\hat{X}}_{t+1}^{j},X_{t}\right)p\left({\hat{X}}_{t+1}^{j}|X_{t}\right)}{1+e^{-Y_{t+1}\Phi \left({\vec{v}}_{t}\right)}}. \end{array}$$

Finally, we obtain the most likely target state $X_{t+1}$ at t+1 with estimated MAP over the *M* samples ${\hat{X}}_{t+1}^{j}$ and its observations ${\hat{Y}}_{t+1}^{j}$ for j=1,…,M, given $X_{t}$,

(12) $$\begin{array}{c} X_{t+1}=\underset{{\hat{X}}_{t+1}^{j},\;1\leq j\leq M}{\mathrm{argmax}}p\left({\hat{X}}_{t+1}^{j}|{\hat{Y}}_{t+1}^{j},X_{t}\right)\\ =\underset{{\hat{X}}_{t+1}^{j},\;1\leq j\leq M}{\mathrm{argmax}}\frac{p\left({\hat{Y}}_{t+1}^{j}|{\hat{X}}_{t+1}^{j},X_{t}\right)p\left({\hat{X}}_{t+1}^{j}|X_{t}\right)}{1+e^{-{\hat{Y}}_{t+1}^{j}\Phi \left({\vec{v}}_{t}\right)}}. \end{array}$$

On the other hand, it is reasonable to infer that the maximizing target state $X_{t+1}$ is very similar to $X_{t}$, which implies $Y_{t+1}\Phi \left({\vec{v}}_{t}\right)\geq 0$, so that $Y_{t+1}\Phi \left({\vec{v}}_{t}\right)\geq {\hat{Y}}_{t+1}^{j}\Phi \left({\vec{v}}_{t}\right)$ for all 1≤j≤M. From this aspect, let ${\tilde{X}}_{t+1}$ be a sample state such that ${\tilde{Y}}_{t+1}\Phi \left({\vec{v}}_{t}\right)\geq 0$ and the solution to the maximization:

(13) $${\tilde{X}}_{t+1}=\underset{{\hat{X}}_{t+1}^{j},\;1\leq j\leq M}{\mathrm{argmax}}p\left({\hat{Y}}_{t+1}^{j}|{\hat{X}}_{t+1}^{j},X_{t}\right)p\left({\hat{X}}_{t+1}^{j}|X_{t}\right).$$

Then, for all 1≤j≤M, we have:

$$\begin{array}{c} \begin{array}{c} p\left({\tilde{Y}}_{t+1}|{\hat{X}}_{t+1},X_{t}\right)p\left({\hat{X}}_{t+1}|X_{t}\right)\\ \;\geq p\left({\hat{Y}}_{t+1}^{j}|{\hat{X}}_{t+1}^{j},X_{t}\right)p\left({\hat{X}}_{t+1}^{j}|X_{t}\right)\cdot \frac{1+e^{-{\tilde{Y}}_{t+1}\Phi \left({\vec{v}}_{t}\right)}}{1+e^{-{\tilde{Y}}_{t+1}\Phi \left({\vec{v}}_{t}\right)}}\\ \;\geq p\left({\hat{Y}}_{t+1}^{j}|{\hat{X}}_{t+1}^{j},X_{t}\right)p\left({\hat{X}}_{t+1}^{j}|X_{t}\right)\cdot \frac{1+e^{-{\tilde{Y}}_{t+1}\Phi \left({\vec{v}}_{t}\right)}}{1+e^{-{\hat{Y}}_{t+1}^{j}\Phi \left({\vec{v}}_{t}\right)}} \end{array} \end{array}$$

for $1+\exp\left(-Y_{t+1}\Phi \left({\vec{v}}_{t}\right)\right)\leq 1+\exp\left(-{\hat{Y}}_{t+1}^{j}\Phi \left({\vec{v}}_{t}\right)\right)$, so that:

$$\frac{p\left({\tilde{Y}}_{t+1}|{\hat{X}}_{t+1},X_{t}\right)p\left({\hat{X}}_{t+1}|X_{t}\right)}{1+e^{-{\tilde{Y}}_{t+1}\Phi \left({\vec{v}}_{t}\right)}}\geq \frac{p\left({\hat{Y}}_{t+1}^{j}|{\hat{X}}_{t+1}^{j},X_{t}\right)p\left({\hat{X}}_{t+1}^{j}|X_{t}\right)}{1+e^{-{\hat{Y}}_{t+1}^{j}\Phi \left({\vec{v}}_{t}\right)}}$$

for all j=1,…,M. This shows that we may regard the denominator $1+e^{-{\hat{Y}}_{t+1}\Phi \left({\vec{v}}_{t}\right)}$ in (12) as a constant for all j=1,…,M.

**Figure 2:**
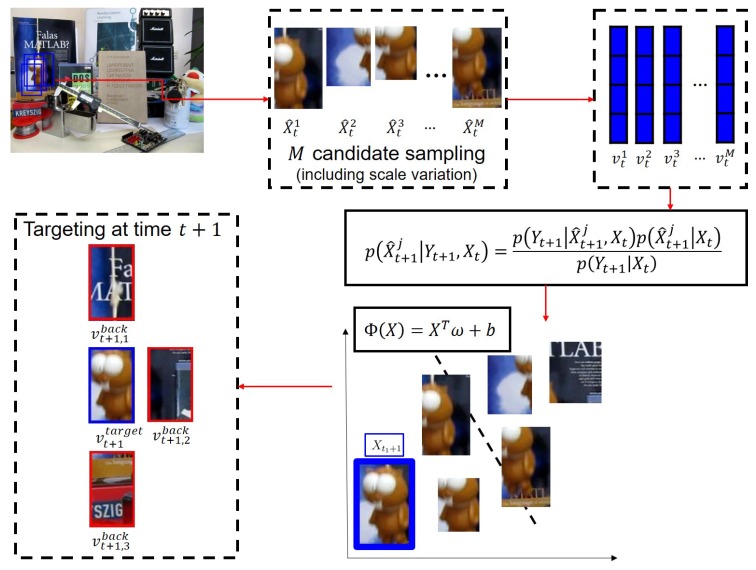
Representation of the target object using structured sparse PCA and deterministic classification between the target object and background image patches.

**Figure 3:**
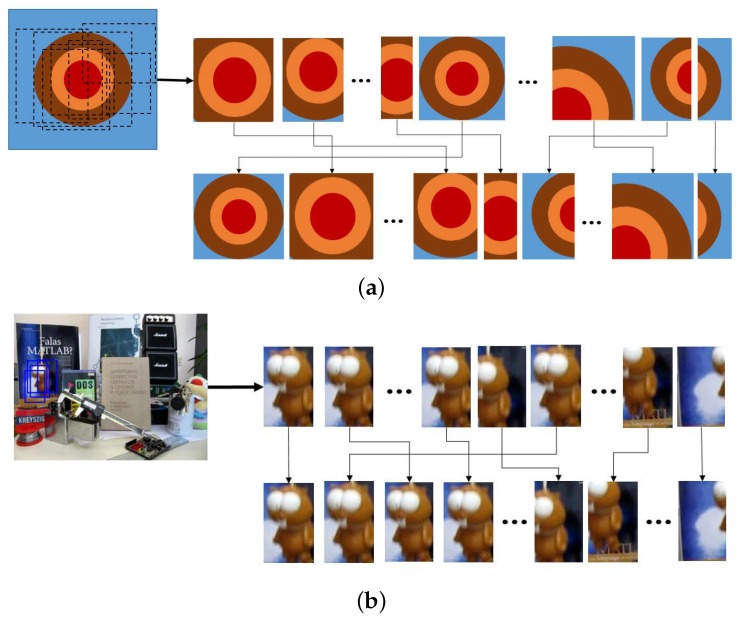
Procedure to find the most similar target object templates using confidence (Equation (9)). (**a**) Typical explanation to find the target object by weighting the scale factor from positive candidate templates to prevent drift, partial occlusion and scaling problems; (**b**) real image-based re-weighting procedure to find similar templates from positive image templates.

Figure 2 shows the steps of how to detect the target object when a new frame comes in. *M* candidate samples are separated into positive and negative labels using $\Phi (\vec{v})$. Usually, the ideal target template contains all of the target features, although there is some background. However, in most cases, a sample with the highest probability tends to contain less background. Figure 3a illustrates this problem. The first row of Figure 3a shows candidate samples sorted without the window size ratio in Equation (9). The ideal candidate sample is located in the fourth. However, the second row, which applied the window size ratio in Equation (9), shows that there is the ideal candidate in the first position. Consequently, we prioritize templates with the same or similar Φ such that larger window sizes are assigned a larger weight, based on the scale information of the last target estimate $X_{t-1}$ (see Equation (9)). Figure 3b illustrates how the result changes when the prioritization is applied.

The proposed motion tracking model is summarized in Algorithm 2.

**Algorithm 2:** Motion tracking model. for $t+1=t_{0}+1$ to the end of the frame sequence
 1.take *M* candidate states ${\left\{{\hat{X}}_{t+1}^{j}\right\}}_{j=1}^{M}∼N\left(X_{t},\vec{\sigma }\right)$around the point $\left(x_{t}^{c},y_{t}^{c}\right)$ 2.build up the *M* descriptors ${\left\{{\vec{v}}_{t+1}^{j}\right\}}_{j=1}^{M}$and their measurements ${\left\{{\hat{Y}}_{t+1}^{j}\right\}}_{j=1}^{M}$ 3.compute the motion model $p({\hat{X}}_{t+1}^{j}|X_{t})$ (Equation (8)) 4.compute the observation model $p({\hat{Y}}_{t+1}^{j}|{\hat{X}}_{t+1}^{j},X_{t})$ by Equation (10) 5.estimate the a posteriori prob. $p({\hat{X}}_{t+1}^{j}|{\hat{Y}}_{t+1}^{j},X_{t})$ using Equation (11) 6.find the most likely target state $X_{t+1}$ by Equation (12) 7.create the target descriptor ${\vec{v}}_{t+1}^{tg}\in R^{s}$ 8.create the background descriptors ${\vec{v}}_{t+1,(a_{x},b_{x})}^{bg}\in R^{s}$ end

Since the appearances of the target may change during tracking, we need to update the classifier Φ every *k* frames by updating the dictionaries as follows.

1. We save the $t_{0}$ target descriptors ${\vec{v}}_{1:t_{0}}^{tg}\in R^{s}$ into a set $F=\{v_{1}^{tg},\ldots ,v_{t_{0}}^{tg}\}$ at time $t=t_{0}$.
2. At every $t>t_{0}$, if $p\left(X_{t}^{j}|Y_{t}^{j},X_{t-1}\right)>\theta_{p}$, we add the target descriptor $v_{t}^{tg}$ and background descriptors ${\vec{v}}_{t,(a_{x},b_{x})}^{bg}$ to *F*. Otherwise, $k_{p}=k_{p}+1$.
3. After every *k* frames, we create the dictionary matrix $D_{w}$ and coefficient matrix $C_{w}$ using the vectors in *F* by applying the structured sparse PCA.
4. Similar to the initiation algorithm, we update Φ using the new $D_{w}$ and $C_{w}$.
5. We check $\Phi (\vec{v})$ for all target descriptors $\vec{v}\in F$ and sort the descriptors according to their values, while keeping the $k_{0}$ largest target descriptors in *F* and deleting the remaining target descriptors and all the background descriptors from *F*.

The update interval $k=k_{0}+k_{p}$ is between the range of $k_{0}$ and $2k_{0}$. Because occlusion frames do not have (whole or partial) target patch, we need to update the dictionary slowly by increasing the value of $k_{0}$.

**Figure 4:**
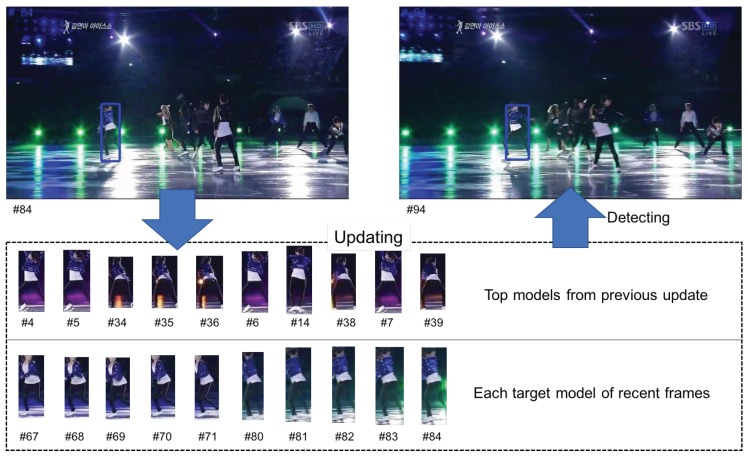
Target models in *F* at the update time and the detection of the target at a later frame.

We continuously update the training dictionaries using the $k_{0}$ prior templates, which have a high probability, from the target as shown in Algorithm 3. This way, if the confidence of the target is high, it will participate in the update continuously. Therefore, the target models with high confidence in the previous update and the target models in recent frames participate in the update. The target models in recent frames keep tracking when the appearance of the target object is almost unchanged, and the target models with high confidence help tracking to not fail when the appearance of the target object changes suddenly. Figure 4 shows the target models in *F* at the update time and the detection of the changed target appearance after the update. In the 84th frame, the top $k_{0}$ target models from previous updates are different from the current target appearance, but show a similar look to the target in the 94th frame. It can be seen that this is more suitable for detecting the changed appearance.

**Algorithm 3:** Dictionary update.  for $t=t_{0}+1$ to the end of the frame sequence   1. if $p\left(X_{t}^{j}|Y_{t},X_{t-1}\right)>\theta_{p}$    1-1. add the target descriptor $v_{t}^{tg}$    and background descriptors ${\vec{v}}_{t,(a_{x},b_{x})}^{bg}$ to *F*   1. else    1-2. $k_{p}=k_{p}+1$  for every *k* frames   2. build the new metrics $D_{w}$ and $C_{w}$ using the vectors in *F*   by structured sparse PCA   3. update classifier Φ using $D_{w}$ and $C_{w}$   4. compute $\Phi (\vec{v})$ for $\vec{v}\in F$   5. keep the $k_{0}$ largest target descriptors in *F*,   and delete the rest descriptor from *F*   6. $k_{p}=0$  end

## 4. Experimental Validation

This section validates the robustness of the proposed method by quantitatively and qualitatively comparing it to current state of the art approaches using the TS-50 public visual object benchmark video sequences (available online: http://cvlab.hanyang.ac.kr/tracker_benchmark/datasets.html (accessed on 8 May 2012)). The benchmark sequences include background clutter (BC), deformation (DEF), fast motion (FM), in-plane rotation (IPR), illumination variation (IV), low resolution (LR), motion blur (MB), occlusion (OCC), out-of-plane rotation (OPR), out-of view (OV) and scale variation (SV). The proposed tracker was implemented in MATLAB on a standard 4-GHz machine with 2 GB RAM. To create the descriptors, we resize all patches to [72, 72] and use the scale-invariant feature transform (SIFT) [50]. The number of samples *M* is set to 600. $t_{0}$ and *r* are set to three and 30, respectively. The $k_{0}$ and $\theta_{p}$ are set to 10 and 0.2, respectively. We also tested the prototype VTD [30], MS [19], MIT [22], SCM [29], Frag [25], IVT [24], TLD [27], Struct [28], and ASLA [11]. The experimental results are compared in Table 2.

The proposed method can be extended to track the target object using the observation model by incorporating various descriptors, and the results are presented in the Supplementary Material. All the MATLAB code and results are available on our web site.

### 4.1. Qualitative Analysis

The public TS-50 video sequences used in the experiments include illumination change, partial occlusion, background clutter, low resolution and pose variations. The proposed structured sparse PCA-based visual object tracking system addresses the main problems by feature optimization and dimensionality reduction.

#### 4.1.1. Significant Occlusion

**Figure 5:**
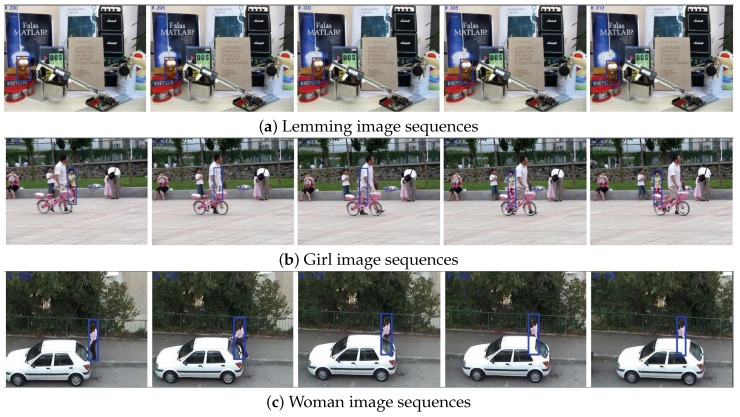
Tracking during partial occlusion and drift.

Heavy occlusion leads to target object tracking drift due to a lack of features, but the learned local structure of the appearance model and online updating prevent the proposed tracker from creating a bias toward part of the target, mitigating the influence of background pixels. Figure 5 shows that although the target object undergoes significant occlusion for a long period, the tracker robustly retains the key appearance structure, reducing the background effect. The Girl sequence in particular shows heavy occlusion from an object with a similar shape to the target object, but the proposed system retains target tracking.

#### 4.1.2. Illumination Change

**Figure 6:**
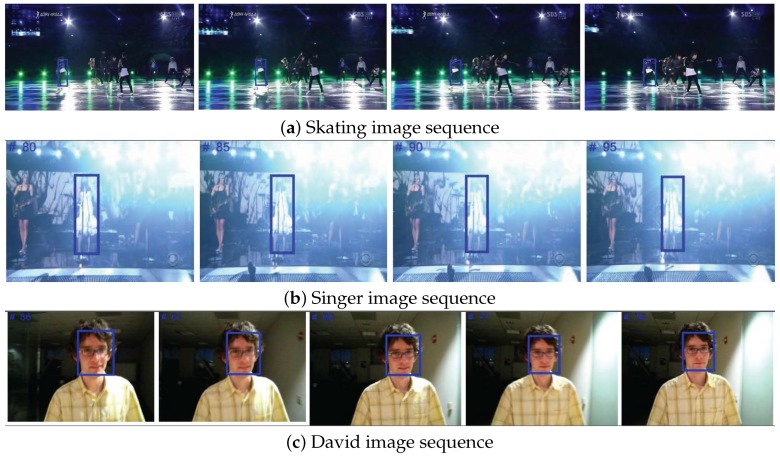
Tracking during illumination changes.

The appearance model using structured sparse representation with an SIFT descriptor is relatively insensitive to illumination changes. Figure 6 shows that although the image sequences include significant illumination changes, the target object remains continuously within the bounding box using the proposed tracking system. Simultaneous update of target images and retention of important structures using the structure sparse PCA method ensure the proposed system continuously tracks the target object even with large illumination changes.

#### 4.1.3. Background Clutter

**Figure 7:**
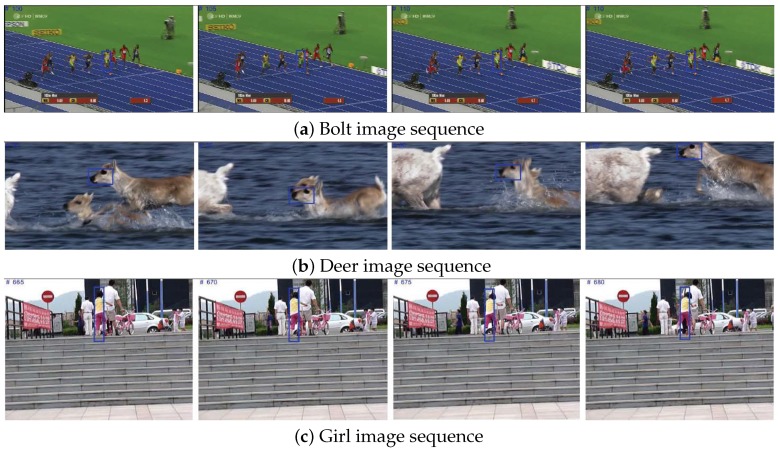
Tracking during background clutter changes.

Discriminative classification of the target object and background images provides clear separation between the target object and background, which have similar color, appearance and motion. Figure 7 shows that the separation of the background and target is very robust against background clutter changes.

### 4.2. Quantitative Analysis

**Figure 8:**
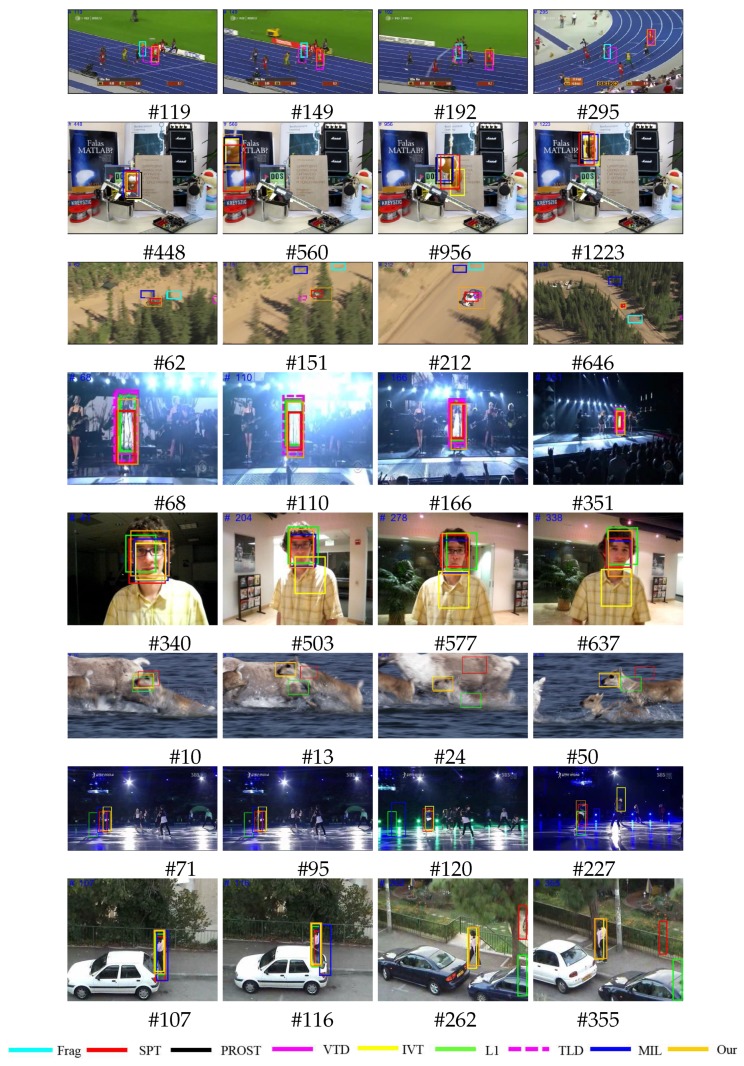
Tracking comparison for the proposed and current state of the art trackers for the Bolt, Lamming, Racecar and Singer image sequences.

We obtained the ground-truth reference values for the eight image sequences, and employed the average of overlap scores (AOS) between the tracking window and ground truth center to quantify the proposed and reference tracker performances [6]. As shown in Table 2, our proposed approach is good for deformation, fast motion, out-of-plane rotation (OPR) and out-of-view (OV), but showed balanced performance per various challenging issues in the visual object tracking. Struct [28] shows a robust performance for various performance test. SCM [29] has good performance in background clutter, illumination variation, occlusion and scale variation because it extracts the features of the target object using sparse representation, but still has variation in the video sequences like fast motion and motion blur. Figure 8 compares the performances for the proposed and current state of the art trackers for the various image sequences. The proposed tracker system tracks the target object under the partial occlusion, drift, background clutter, scale and pose variation challenges.

## 5. Conclusions

We proposed a structured sparse PCA-based visual object tracking incorporating initialization, motion tracking and online dictionary learning and update. In the initialization stage, a discriminative classifier was applied to target object and background image template coefficients extracted from the structured sparse PCA. The best candidate samples were selected by jointly evaluating the appearance distance and learned classifier. Online dictionary learning was based on a sparse representation appearance model where the dictionary and classifier were continuously updated. The structured sparse PCA provided dimensionality reduction of high dimensional descriptors, while retaining the structure of the appearance model.

We experimentally evaluated the effectiveness of the proposed tracking system by comparing with the twelve current state of the art trackers using eight publicly available benchmark image sequences. The proposed method performed favorably against all current trackers and was able to handle all the various tracking challenge scenarios. Quantitative and qualitative comparison of the outcomes from the challenging image sequences validated the effectiveness and robustness of the proposed algorithm.

Thus, exploiting a linear combination of key structure features using structured sparse PCA is a robust method to track target objects through illumination, partial occlusion and background clutter changes, because the structure of the appearance model effectively estimates the similarity between the target object and candidates.

## Figures and Tables

**Table 1:** Notations and symbols.

Symbol	Description
$I_{t}$	Frame at time *t*
$X_{t}\in R^{2}\times R\times R$	State variable $X_{t}=\left({\vec{x}}_{t}^{c},w_{t}^{sx},h_{t}^{sy}\right)$
$Y_{t}\in \left\{-1,1\right\}$	Observation variable
${\vec{x}}^{c}=\left(x^{c},y^{c}\right)\in R^{2}$	Location vector in the state variable $X_{t}$
${\vec{v}}^{tg}\in R^{s}$	Target descriptor vector
${\vec{v}}^{bg}\in R^{s}$	Background descriptor vector
$\vec{p}$	Patch image
$\vec{d}$	Column vectors of *D*
$\vec{c}$	Column vectors of *C*
*V*	Feature descriptor
*D*	Feature dictionary
*C*	Feature coefficient matrices
Φ	Support vector machine classifier
$\vec{\sigma }$	4×4 Diagonal covariance matrix
$N(X_{t};X_{t-1},\vec{\sigma })$	Multivariate normal distribution
*F*	Set of target descriptors
*p*	Probability function
*s*	Dimension of descriptors
*r*	Number of dictionary vectors
κ	Number of background descriptors ${\vec{v}}^{bg}$
*k*	Number of vectors after updating
*t*	Time variable
$a_{x}$	Real number −1 or 1 related to width size
$b_{y}$	Real number −1 or 1 related to height size
$w_{t}^{sx}$	Width (*x*-axis) size of patch
$h_{t}^{sy}$	Height (*y*-axis) size of patch
≈	Approximately equal
∝	Proportional to
$\cdot^{T}$	Transpose operator

**Table 2:** Average of overlap score of the proposed tracker and several current state of the art trackers ((BC), deformation (DEF), fast motion (FM), in-plane rotation (IPR), illumination variation (IV), low resolution (LR), motion blur (MB), occlusion (OCC), out-of-plane rotation (OPR), out-of view (OV) and scale variation (SV)). The top two methods for each dataset are highlighted in red and blue, respectively. VTD, visual tracking decomposition; MS, mean-shift; MIL, multiple instance learning; SCM, sparse collaborative appearance; Frag, fragment-based; TLD, tracking-learning-detection.

	All	BC	DEF	FM	IPR	IV	LR	MB	OCC	OPR	OV	SV
Proposed	58.50	60.19	58.78	56.74	56.22	52.03	60.86	58.96	55.00	57.13	56.51	52.33
VTD [30]	49.3	55.1	46.2	41.7	50.2	53.7	47.1	43.5	52.3	53.7	51.5	48.9
MS [19]	35.6	36.7	32.8	40.5	36.8	34.6	28.4	41.2	37.4	37.3	41.0	36.0
MIL [22]	45.9	48.6	45.7	44.1	45.7	47.1	43.5	43.7	47.6	48.9	52.7	44.5
SCM [29]	54.4	61.3	51.5	42.8	51.8	61.1	61.7	45.2	56.8	57.0	56.4	55.8
Frag [25]	44.2	46.1	41.8	44.8	43.3	42.6	42.6	46.1	46.6	46.1	50.1	44.2
IVT [24]	46.4	51.6	40.5	37.3	46.4	51.2	55.8	41.3	49.3	49.0	52.3	47.1
TLD [27]	46.8	48.3	37.4	44.6	48.9	46.7	53.3	51.0	45.2	46.0	50.2	47.1
Struct [28]	57.5	59.3	52.4	55.6	57.0	59.0	59.1	59.9	55.9	57.3	58.9	57.8
ASLA [11]	53.2	59.2	50.5	42.0	52.1	59.6	59.3	44.6	56.0	56.3	55.3	54.0

## References

[1] Trucco E., Plakas K. (2006). Video tracking: A concise survey. IEEE J. Ocean. Eng..

[2] Yilmaz A., Javed O., Shah M. (2006). Object tracking. ACM Comput. Surv..

[3] Jalal A.S., Singh V. (2012). The State-of-the-Art in Visual Object Tracking. Informatica.

[4] Smeulders A.W.M., Chu D.M., Cucchiara R., Calderara S., Dehghan A., Shah M. (2014). Visual tracking: An experimental survey. IEEE Trans. Pattern Anal. Mach. Intell..

[5] Wu Y., Lim J., Yang M.H. Online object tracking: A benchmark. Proceedings of the IEEE Computer Society Conference on Computer Vision and Pattern Recognition.

[6] Wu Y., Lim J., Yang M.H. (2015). Object tracking benchmark. IEEE Trans. Pattern Anal. Mach. Intell..

[7] Beymer D., McLauchlan P., Coifman B., Malik J. A real-time computer vision system for measuring traffic parameters. Proceedings of the IEEE Computer Society Conference on Computer Vision and Pattern Recognition.

[8] Li X., Hu W., Shen C., Zhang Z., Dick A., van den Hengel A. (2013). A Survey of Appearance Models in Visual Object Tracking. ACM Trans. Intell. Syst. Technol..

[9] Chen F., Wang Q., Wang S., Zhang W., Xu W. (2011). Object tracking via appearance modeling and sparse representation. Image Vis. Comput..

[10] Bai T., Li Y.F. (2012). Robust visual tracking with structured sparse representation appearance model. Pattern Recognit..

[11] Jia X., Lu H., Yang M.H. Visual tracking via adaptive structural local sparse appearance model. Proceedings of the IEEE Computer Society Conference on Computer Vision and Pattern Recognition.

[12] Rubinstein R., Bruckstein A.M., Elad M. (2010). Dictionaries for sparse representation modeling. Proc. IEEE.

[13] Sadeghi M., Babaie-Zadeh M., Jutten C. (2013). Dictionary learning for sparse decomposition: A novel approach. IEEE Signal Process. Lett..

[14] Huang T. Linear spatial pyramid matching using sparse coding for image classification. Proceedings of the 2009 IEEE Conference on Computer Vision and Pattern Recognition.

[15] Henrigues J.F., Caseiro R., Martins P., Batista J. (2015). High-Speed Tracking with Kernelized Correlation Filters. IEEE Trans. Pattern Anal. Mach. Intell..

[16] Birchfield S.T. KLT: An Implementation of the Kanade-Lucas-Tomasi Feature Tracker. https://cecas.clemson.edu/~stb/klt/.

[17] Kalman R.E. (1960). A new approach to linear filtering and prediction problems. J. Basic Eng..

[18] Ramos J.A. (1990). A kalman-tracking filter approach to nonlinear programming. Comput. Math. Appl..

[19] Comaniciu D., Ramesh V., Meer P. Real-time tracking of non-rigid objects using mean shift. Proceedings of the IEEE Conference on Computer Vision and Pattern Recognition.

[20] Allen J.G., Xu R.Y.D., Jin J.S. (2006). Object Tracking Using CamShift Algorithm and Multiple Quantized Feature Spaces. Reproduction.

[21] Khan Z., Balch T., Dellaert F. (2004). An MCMC-Based Particle Filter for Tracking Multiple Interacting Targets. European Conference on Computer Vision.

[22] Babenko B., Yang M.H., Belongie S.J. Visual tracking with online multiple instance learning. Proceedings of the IEEE Conference on Computer Vision and Pattern Recognition.

[23] Maraghi T.F.E., Fleet D.J., Jepson A.D. Robust online appearance models for visual tracking. Proceedings of the the IEEE Conference on Computer Vision and Pattern Recognition.

[24] Ross D.A., Lim J.W., Lin R.S., Yang M.H. (2008). Incremental learning for robust visual tracking. Int. J. Comput. Vis..

[25] Srikrishnan V., Nagaraj T., Chaudhuri S. Fragment based tracking for scale and orientation adaptation. Proceedings of the Indian Conference on Computer Vision, Graphics and Image Processing.

[26] Kalal Z., Matas J., Mikolajczyk K. P-N learning: Bootstrapping. Proceedings of the IEEE Computer Society Conference on Computer Vision and Pattern Recognition.

[27] Kalal Z., Mikolajczyk K., Matas J. (2012). Tracking-learning-detection. IEEE Trans. Pattern Anal. Mach. Intell..

[28] Hare S., Saffari A., Torr P.H.S. Struck: Structured output tracking with kernels. Proceedings of the International Conference on Computer Vision.

[29] Zhong W., Lu H., Yang M.H. (2014). Robust object tracking via sparse collaborative appearance model. IEEE Trans. Image Process..

[30] Kwon J., Lee K.M. Visual tracking decomposition. Proceedings of the IEEE Computer Society Conference on Computer Vision and Pattern Recognition.

[31] Bao C., Wu Y., Ling H., Ji H. Real time robust L1 tracker using accelerated proximal gradient approach. Proceedings of the IEEE Computer Society Conference on Computer Vision and Pattern Recognition.

[32] Zhang T., Liu S., Xu C., Yan S., Ghanem B., Ahuja N., Yang M.-H. Structural Sparse Tracking. Proceedings of the 2015 IEEE Conference on Computer Vision and Pattern Recognition (CVPR).

[33] Zhang T., Ghanem B., Liu S., Ahuja N. (2017). Robust Visual Tracking via Structured Multi-Task Sparse Learning. Int. J. Comput. Vis..

[34] Chen Z., You X., Zhong B., Li J. (2017). Dynamically Modulated Mask Sparse Tracking. IEEE Trans. Cybern..

[35] Wang N., Yeung D.-Y. Learning a deep compact image representation for visual tracking. Proceedings of the Neural Information Processing Systems.

[36] Hong S., You T., Kwak S., Han B. Online tracking by learning discriminative saliency map with convolutional neural network. Proceedings of the International Conference on Machine Learning.

[37] Zhang D., Maei H., Wang X., Fang Y. (2017). Deep Reinforcement Learning for Visual Object Tracking. arXiv.

[38] Nam H., Han B. Learning Multi-Domain Convolutional Neural Networks for Visual Tracking. Proceedings of the IEEE Conference on Computer Vision and Pattern Recognition (CVPR).

[39] Wang L., Ouyang W., Wang X., Lu H. STCT: Sequentially Training Convolutional Networks for Visual Tracking. Proceedings of the 2016 IEEE Conference on Computer Vision and Pattern Recognition (CVPR).

[40] Yang F., Lu H., Yang M.-H. (2014). Robust superpixel tracking. IEEE Trans. Image Process..

[41] Donoho D.L. (2006). Compressed sensing. IEEE Trans. Inf. Theory.

[42] Candes E., Wakin M. (2008). An introduction to compressive sensing. IEEE Signal Process. Mag..

[43] Cheng H. (2015). Sparse Representation, Modeling and Learning in Visual Recognition—Theory, Algorithms and Applications.

[44] Kreutz-Delgado K., Murray J.F., Rao B.D., Engan K., Lee T.-W., Sejnowski T.J. (2003). Dictionary learning algorithms for sparse representation. Neural Comput..

[45] Wright J., Ma Y., Mairal J., Sapiro G., Huang T.S., Yan S. (2010). Sparse representation for computer vision and pattern recognition. Proc. IEEE.

[46] Elhamifar E., Vidal R. Robust classification using structured sparse representation. Proceedings of the IEEE Conference on Computer Vision and Pattern Recognition (CVPR).

[47] Bronstein A.M., Sprechmann P., Sapiro G. (2012). Learning efficient structured sparse models. arXiv.

[48] Varshney K.R., Çetin M.J.W., Fisher J.W., Willsky A.S. (2008). Sparse representation in structured dictionaries with application to synthetic aperture radar. IEEE Trans. Signal Process..

[49] Jenatton R., Obozinski G., Bach F.R. Structured sparse principal component analysis. Proceedings of the Thirteenth International Conference on Artificial Intelligence and Statistics (AISTATS).

[50] Lowe D.G. (2004). Distinctive Image Features from Scale-Invariant Keypoints. Int. J. Comput. Vis..

